# Advances in Carrageenases: Molecular Engineering, Immobilization Techniques, and Biomedical Potential of Carrageenan Oligosaccharides

**DOI:** 10.3390/md24080287

**Published:** 2026-08-21

**Authors:** Qiangzhuang Xie, Jiahong Lan, Yanhong Chen, Zhipeng Li, Lijun Li, Zedong Jiang, Hui Ni, Yanbing Zhu

**Affiliations:** 1 College of Ocean Food and Biological Engineering, Jimei University, Xiamen 361021, China; xieqiangzhuang2071@163.com (Q.X.); ljh18120730086@163.com (J.L.);; 2 Fujian Provincial Key Laboratory of Food Microbiology and Enzyme Engineering, Xiamen 361021, China

**Keywords:** carrageenases, catalytic mechanism, molecular engineering, carrageenan oligosaccharides

## Abstract

Carrageenan is a sulfated polysaccharide extracted from red algae, widely utilized in the food and pharmaceutical industries due to its unique gelling properties and biological activities. However, its high molecular weight and low solubility limit certain high-value applications. Carrageenases specifically degrade carrageenan to produce low-molecular-weight oligosaccharides with excellent bioactivities, thereby overcoming this bottleneck. This review systematically summarizes the microbial sources of carrageenases and the classification characteristics of their three types (κ, ι, and λ) within the GH16, GH82, and GH150 families. Furthermore, it highlights recent advances in molecular engineering strategies, including site-directed mutagenesis and non-catalytic domain fusion, as well as enzyme immobilization techniques utilizing carriers such as magnetic nanoparticles and metal–organic frameworks, which significantly enhance thermostability, catalytic efficiency, and reusability. Additionally, the potential applications of carrageenan oligosaccharides in biomedical fields, including antioxidant, immunomodulatory, antitumor, and anti-inflammatory activities, are thoroughly discussed. This review provides a theoretical reference for the rational design and industrial application of carrageenases, while also offering new insights for the high-value development of algal resources.

## 1. Introduction

**Figure 1:**
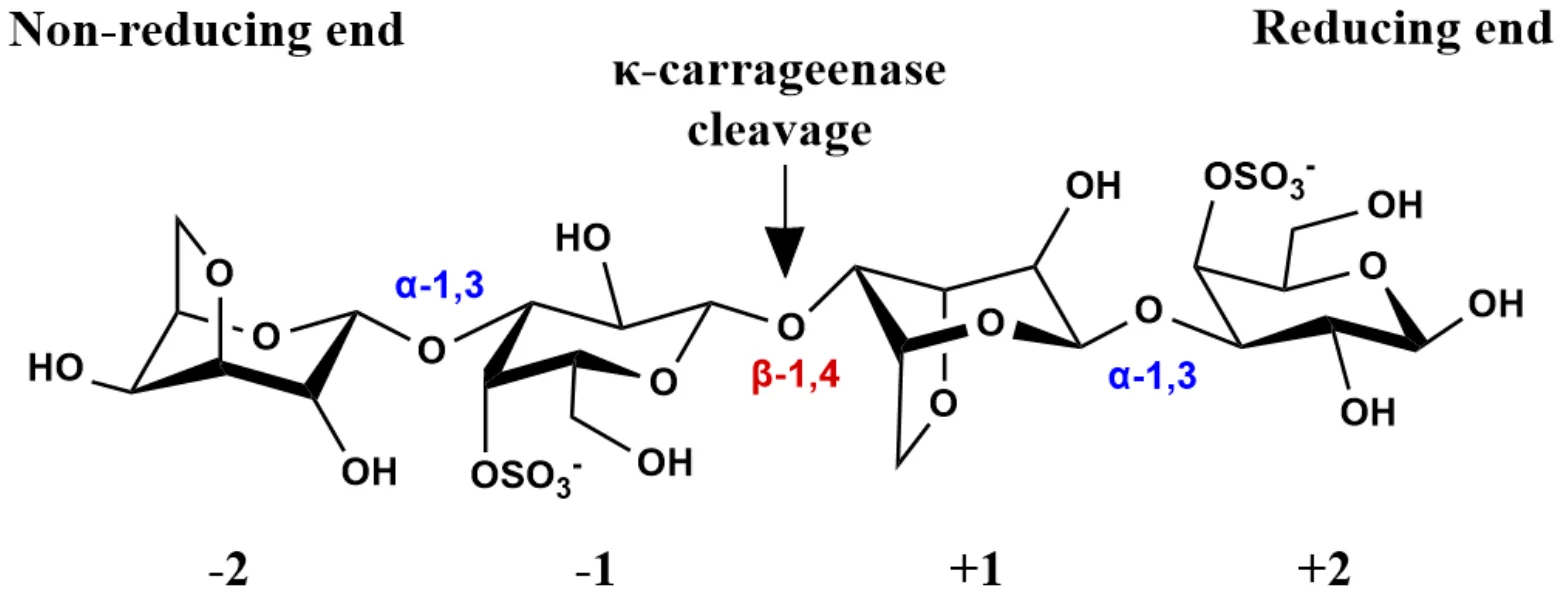
Structure of κ-neocarratetraose with reducing and non-reducing ends indicated [3].

Carrageenan is a class of sulfated linear polysaccharides extracted from red algae (e.g., *Chondrus* and *Eucheuma*). Its unique chemical structure and bioactivity have led to its widespread application in the food, pharmaceutical, and biomaterial industries [1,2]. Structurally, carrageenan consists of repeating disaccharide units. Typically, these units are composed of alternating β-1,4-linked D-galactose (G unit) and α-1,3-linked 3,6-anhydro-D-galactose (A unit) residues, or consist entirely of two D-galactose (G unit) residues. The tetrasaccharide repeating unit, κ-neocarratetraose, is depicted in Figure 1 [3]. The nomenclature of carrageenans follows a Greek letter system (κ, ι, λ) originally proposed by Knutsen et al. (1994), which designates idealized repeating disaccharide structures based on the number and position of sulfate ester groups and the presence of 3,6-anhydrogalactose [4]. It should be emphasized, however, that naturally extracted carrageenans are rarely homogeneous, they typically occur as molecular hybrids containing multiple disaccharide motifs within the same polymer chain [5,6]. Variable degrees of sulfation occur at the G and A units, giving rise to different types of carrageenan [7].

According to market statistics, the global carrageenan market was valued at approximately USD 1.1 billion in 2024 and is projected to reach USD 1.4 billion by 2030. Annual global production exceeds 60,000 metric tons. Carrageenan-containing seaweeds are the largest contributors to both food (62%) and non-food (55%) hydrocolloid applications [8]. Bulk carrageenan is traded at approximately USD 9 per kilogram. By contrast, carrageenan oligosaccharides command substantially higher prices, averaging USD 123 per kilogram with gross profit margins of approximately 50%. The global oligosaccharide market was valued at USD 8.35–145 million in 2024 and is expected to grow at a compound annual rate of 5.4–10.8% through 2031–2033. This considerable value gradient—from bulk polysaccharide to bioactive oligosaccharides—provides a strong economic driver for developing efficient carrageenase-based production technologies.

**Figure 2:**
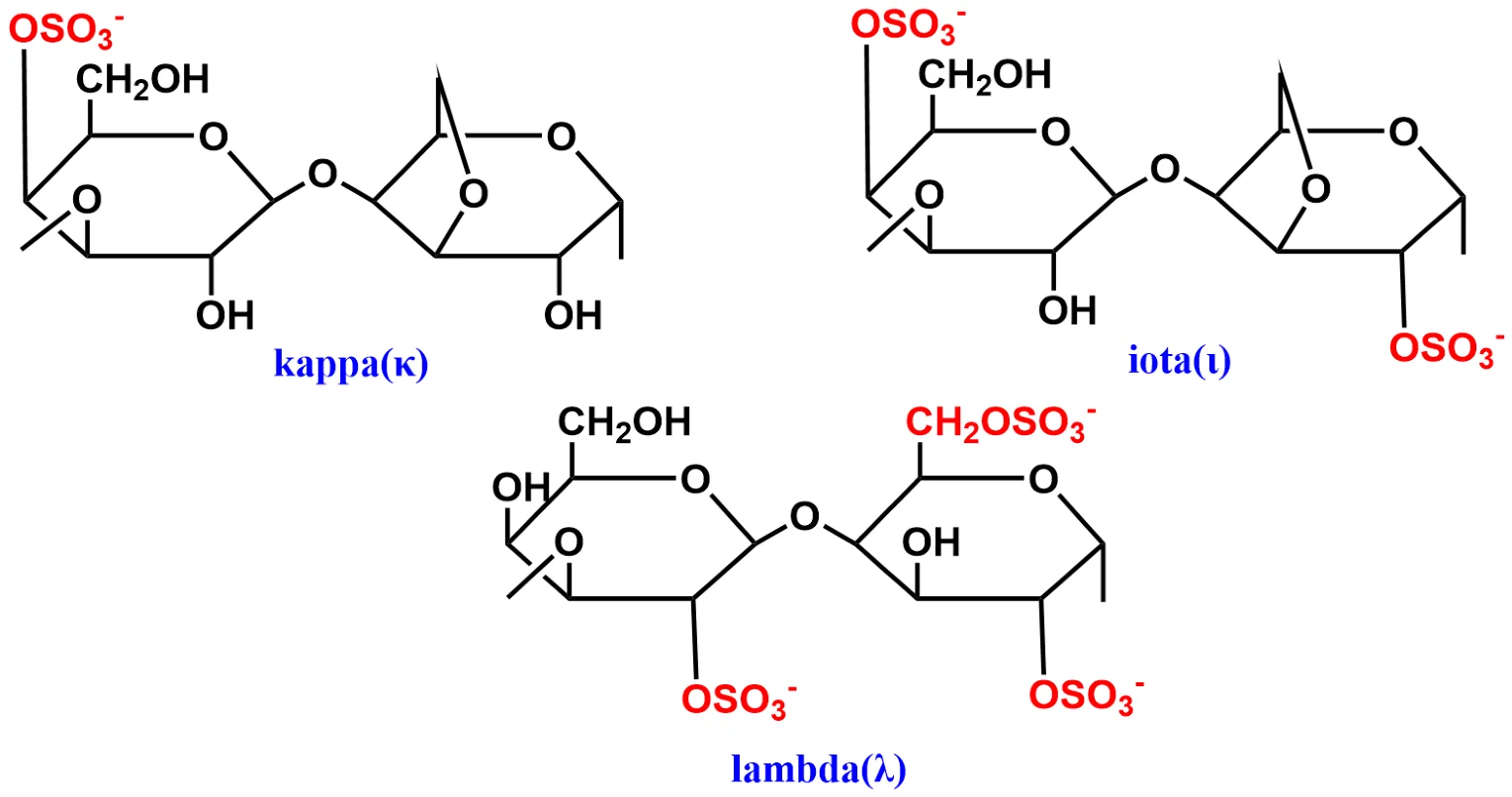
Structures of disaccharide repeating units of κ-, ι-, and λ-carrageenans [9].

Carrageenan can be classified based on its sulfation pattern and the presence or absence of 3,6-anhydrogalactose (3,6-AG) (Figure 2) [9]. The commercially common types mainly include κ-, ι-, and λ-carrageenan [10]. κ-Carrageenan is primarily derived from *Kappaphycus alvarezii* (formerly *Eucheuma cottonii*), ι-carrageenan from *Eucheuma denticulatum*, and λ-carrageenan from *Chondrus crispus* and *Gigartina* species. The structural differences among these types determine their functional properties and diverse industrial applications [11]. At the structural level, the substitution position of sulfate groups and the content of 3,6-AG in different isomers directly influence their gelling ability. Among them, the κ-type forms strong, relatively rigid gels and is therefore the most widely used in the food industry [12,13]. The presence of 3,6-AG in the κ- and ι-types enhances molecular chain rigidity, promotes helix formation and double-helix aggregation, thereby facilitating gel network construction. In contrast, the λ-type has a higher sulfation density, which endows it with stronger polyanionic characteristics and tends to induce strong electrostatic interactions, but typically inhibits gelation [14]. κ-Carrageenan forms K^+^-dependent brittle gels through double-helix aggregation [15]. ι-Carrageenan produces elastic gels via the formation of helix bundles in the presence of Ca^2+^ [16]. In contrast, λ-carrageenan, due to its extended helical conformation and strong electrostatic repulsion between sulfate groups, generally lacks gelling ability but can exhibit high viscosity [17]. The overall sulfation degree of carrageenan follows the trend λ > ι > κ. A higher sulfation degree is generally associated with stronger hydrophilicity and higher anticoagulant activity, but reduces thermal stability [17]. In marine algae, carrageenan enhances the structural stability of the cell wall through electrostatic crosslinking mediated by sulfate groups, and also acts as a defensive molecule that inhibits pathogen adhesion via steric hindrance [18]. Moreover, oligosaccharides derived from κ/ι-carrageenan by enzymatic degradation exhibit antiviral and immunomodulatory activities, suppressing HSV-1 infection through a receptor-blocking mechanism [19,20]. In contrast, the high sulfation degree of λ-carrageenan confers heparin-like anticoagulant properties, although its clinical application still requires strict dosage control to mitigate the risk of potential cytotoxicity [9]. Among the preparation methods for carrageenan oligosaccharides, chemical hydrolysis and enzymatic degradation are the most widely employed. However, chemical hydrolysis of carrageenan, typically conducted under acidic conditions, causes nonspecific structural damage including desulfation, ring opening, and formation of undesirable by-products such as monosaccharides and furfural [21]. Enzymatic hydrolysis using carrageenases operates under milder conditions, affords higher product specificity, and better preserves sulfate groups essential for bioactivity [22]. However, enzymatic processes entail higher production costs, whereas chemical methods are more cost-effective at scale but yield heterogeneous product mixtures with poor batch-to-batch consistency. Acid hydrolysis also raises regulatory concerns, as harsh treatment can generate poligeenan, a degraded carrageenan product that is prohibited in food systems worldwide. Recent strategies combining mild acid pretreatment with subsequent enzymatic refining may offer a pragmatic compromise between cost and product quality [22].

Carrageenases are enzymes capable of cleaving the internal β-1,4 glycosidic bonds within carrageenan polymers, releasing carrageenan oligosaccharides of varying lengths composed of repeating disaccharide units. The degradation of κ-carrageenan to yield κ-carrageenan oligosaccharides can significantly improve properties such as poor solubility and low bioavailability [23]. Furthermore, carrageenan enzymatic degradation products hold great potential for biomedical and physiological applications, including antioxidant [24], antitumor [25], immunomodulatory [26], antiviral [27], antibacterial [28], and anti-inflammatory activities [29]. Consequently, carrageenases have become essential tools for the production of high-value-added carrageenan oligosaccharides. This article provides a comprehensive summary of the diversity, classification, and enzymatic properties of carrageenases derived from different microbial sources, with a focus on elucidating the correspondence between their structural features and catalytic mechanisms. The accumulation of this knowledge offers critical theoretical support for the rational design and industrial application of carrageenases.

## 2. Sources, Types, Properties, and Degradation Patterns of Carrageenases

Carrageenases are microbial enzymes primarily produced by marine bacteria and archaea, with a wide range of sources including microorganisms and marine animals. The main bacterial genera capable of secreting carrageenases include *Pseudoalteromonas*, *Pseudomonas*, *Shewanella*, *Vibrio*, *Cellulophaga*, *Cytophaga*, *Tamlana*, *Microbulbifer*, *Alteromonas*, *Pedobacter*, *Zobellia*, among which *Pseudoalteromonas*, *Vibrio*, and *Alteromonas* are the predominant sources [30]. Bacteria utilize carrageenases as enzymatic tools to degrade carrageenan for invasion, and the degradation products can sometimes serve as inducers for further carrageenase production [31]. Additionally, carrageenase activity has been detected in the intestinal secretions of herbivorous fishes [32]. Carrageenases are classified into three types—κ, ι, and λ—corresponding to the CAZy families GH16, GH82, and GH150, respectively [33,34,35]. High-resolution structural studies have revealed that GH16 enzymes adopt a β-barrel fold, GH82 enzymes exhibit a right-handed parallel β-helix, while GH150 enzymes, though less studied, display a distinctive domain organization [34,35,36].

In recent years, the identification and characterization of novel carrageenases from carrageenan-hydrolyzing microorganisms has attracted significant attention [37]. Table 1, Table 2, Table 3 and Table 4 summarize the sources and key properties of carrageenases. Among these, the molecular weights of carrageenases are typically determined by protein ladder migration on sodium dodecyl sulfate-polyacrylamide gel electrophoresis (SDS-PAGE). In most cases, purified carrageenases appear as a single band [38]. Interestingly, three isoforms of κ-carrageenase have been identified in *Cytophaga* sp., which were detected as more than one protein band on SDS-PAGE [39]. According to the reports, the molecular weights of carrageenases across different microbial species range from 27.6 kDa to 128 kDa [37].

Studies have shown that the optimal pH for carrageenases ranges between 7.2 and 8.0, close to seawater pH value [40,41]. However, exceptions exist. For example, κ-carrageenases from *Zobellia* sp. ZM-2 [42], *Cellulophaga lytica* N5-2 [43], and *Pseudoalteromonas tetraodonis* JAM-K142 [44] exhibit optimal activity at pH 6.0, 7.0, and 8.8, respectively. Notably, a κ-carrageenase isolated from *Pseudomonas elongala* MTCC5261 displayed optimal activity under both acidic (pH 5.6) and alkaline (pH 7.7) conditions [45]. The optimal temperature range for carrageenases is 30–55 °C, but approximately 7% of these enzymes (e.g., those derived from polar strains) exhibit maximal activity below 25 °C [35], while 11% (e.g., from deep-sea hydrothermal vent strains) show maximal activity above 70 °C [46]. For instance, Jiang et al. reported that the thermostable κ-carrageenase MtKC16A reaches peak activity at 80 °C and maintains stability at 100 °C [47]. An ι-carrageenase from *Wenyingzhuangia fucanilytica* exhibits maximal activity at a low temperature of 25 °C [48]. And a λ-carrageenase from *Bacillus* sp. Lc50-1 shows maximal activity at a high temperature of 75 °C [46].

Carrageenase activity is influenced by certain cations due to enzyme conformational changes and substrate alterations. Carrageenases are typically activated in the presence of monovalent metal ions such as Na^+^ and K^+^. In contrast, some heavy metal ions including Co^2+^, Zn^2+^, Mn^2+^, Cu^2+^, Pb^2+^, Hg^2+^, and Ag^+^ can inhibit carrageenase activity [49,50,51]. It has been reported that reducing agents such as DL-dithiothreitol (DTT) can enhance the activity of κ-carrageenase isolated from *Zobellia* sp. ZM-2. This effect is attributed to DTT’s protective action on cysteine residues, which maintains enzyme conformational integrity and stability [42]. The chelating agent ethylenediaminetetraacetic acid (EDTA) exhibits inhibitory effects on some κ-carrageenases derived from *Tamlana* sp. HC [52], *Pseudoalteromonas* sp. QY203 [53], and *Vibrio* sp. NJ-2 [54].

The structural diversity of carrageenan requires specific enzymes to recognize the linear structure and maintain the native configuration of the backbone. Accordingly, enzymes that specifically act on κ-, ι-, and λ-carrageenan are designated as κ-carrageenase (EC 3.2.1.83), ι-carrageenase (EC 3.2.1.157), and λ-carrageenase (EC 3.2.1.162), respectively. Different types of carrageenases are endohydrolases that cleave internal β-1,4 linkages rather than degrading from the termini, releasing even-numbered oligosaccharides. However, the enzymatic degradation products of κ- and ι-carrageenases commonly include oligosaccharides with different degrees of polymerization (DP) such as DP2 and DP4, indicating that they hydrolyze the polysaccharide chains in a processive manner [34,38]. In contrast, λ-carrageenase produces oligosaccharides with higher degrees of polymerization, suggesting that its degradation pattern differs from that of κ-/ι-carrageenases [35]. The diversity in the degree of polymerization of enzymatic products is determined jointly by the heterogeneity of the carrageenan primary structure and the stereochemistry of the glycosidic linkages [36]. Moreover, the carrageenan oligosaccharides obtained via enzymatic cleavage exhibit more uniform molecular weights than those obtained by the acid hydrolysis [55].

The degree of polymerization (DP) of carrageenan oligosaccharides is a critical determinant of their biological functions and application potential. However, the optimal DP for a given bioactivity varies considerably depending on the carrageenan type and the specific molecular target. Specifically, κ-neocarrabiose (DP2) exhibits stronger cytotoxicity and pro-apoptotic effects against LM2 tumor cells [25]. By contrast, the heterogeneous κ/ι-hexasaccharide (DP6) demonstrates superior reactive oxygen species scavenging activity in LPS-induced RAW264.7 macrophages [56]. In the ι-series, the tetrasaccharide (DP4) has been shown to ameliorate insulin resistance in high-fat-high-sucrose diet-fed mice [57]. This effect is mediated by upregulating glucagon-like peptide-1 (GLP-1) and inhibiting mitochondrial apoptosis pathways in pancreatic β cells. Meanwhile, ι-DP8 exhibits prominent anti-inflammatory activity by suppressing TNF-α, IL-6, and nitric oxide production in the same macrophage model [31]. For λ-carrageenan oligosaccharides, a direct comparative study demonstrated that λ-neocarrabiose (DP2) exerts significantly stronger anti-inflammatory activity than λ-neocarraoctaose (DP8) [58]. However, λ- neocarraheptaose (DP7) displays the most potent basic fibroblast growth factor-binding affinity among DP2–DP8 oligosaccharides [59]. This observation suggests that higher DP may be advantageous for certain protein-binding interactions. Collectively, these findings indicate that no single DP universally confers superior bioactivity. Rather, the structure–activity relationship is target-dependent, necessitating precise DP control for specific applications. From an industrial perspective, the capacity to generate oligosaccharides with defined DP profiles through enzyme engineering is essential for ensuring product consistency and meeting regulatory requirements. This capability also enables high-value applications in pharmaceuticals, nutraceuticals, and cosmetics. Thus, controlling enzyme product specificity to produce oligosaccharides with tailored DP distributions represents a strategic imperative for the valorization of carrageenan resources. (Table 1, Table 2, Table 3 and Table 4).

**Table 1:** Sources and biochemical properties of κ-carrageenases from *Pseudoalteromonas* species.

Organism	Optimal pH	Optimal Temperature (°C)	Kinetic Parameters	Thermostability	pH Stability	Hydrolysis Products	Reference
*Pseudoalteromonas tetraodonis* JAM-K142	8.8	30	*K*_m_ = 0.869 mg/mL; *V*_max_ = 66.4 µmol min^−1^ mg^−1^	—	—	—	[44]
*Pseudoalteromonas* sp. QY203	7.2	45	—	40% residual activity at 50 °C for 1 h	6.0 to 9.0	DPs 2 and 4	[53]
*Pseudoalteromonas porphyrae* LL-1	8.0	55	*K*_m_ = 4.4 mg/mL; *V*_max_ = 66.4 mg min^−1^ mL^−1^	50% residual activity at 40 °C for 1 h	6.0 to 9.0	—	[60]
*Pseudoalteromonas* WZUC10	7.5	30	*K*_m_ = 0.56 mg/mL; *k*_cat_ = 125 s^−1^	completely inactivated at 7.5 min at 70 °C	—	DP 4	[61]
*Pseudoalteromonas* sp. AJ5-13	8.0	55	*K*_m_ = 9.8 mg/mL	5% residual activity at 50 °C for 30 min	6.5 to 9.0	DPs 2, 4, 6, 8 and 10	[62]
*Pseudoalteromonas carrageenovora* QY203	7.0	55	—	90% residual activity at 40 °C for 1 h	More than 80% activity at 4 °C for 24 h at pH 3.6–10.0	DPs 2 and 4	[63]
*Pseudoalteromonas porphyrae* LL1	8.0	40	—	20% residual activity at 45 °C for 1 h	70% residual activity at pH 11.0 and 4 °C for 12 h	DPs 2 and 4	[64]
*Pseudoalteromonas* sp. ZDY3	8.0	45	*K*_m_ = 3.67 mg/mL; *K*_m_/*k*_cat_ = 53.0 mL mg^−1^ s^−1^	80% residual activity at 30 °C for 15 d	80% activity in pH 4.5–10.5 for 48 h	DPs 2 and 4	[65]

Note: Original values are retained due to differing units reported in the source literature. “—” indicates data not reported in the original publication.

**Table 2:** Sources and biochemical properties of κ-carrageenases from other genera.

Organism	Optimal pH	Optimal Temperature (°C)	Kinetic Parameters	Thermostability	pH Stability	Hydrolysis Products	Reference
*Zobellia* sp. ZM-2	6.0	39	*K*_m_ = 0.84 mg/mL; *V*_max_ = 2.92 U/mL	50% residual activity at 40 °C for 1 h	remained 85% activity at 20 °C for 2 h at pH 6.0–8.0	DPs 4 and 6	[42]
*Cellulophaga lytica* strain N5-2	7.0	35	*K*_m_ = 1.647 mg/mL; *V*_max_ = 8.7 µmol min^−1^ mg^−1^	25% residual activity at 50 °C for 1 h	—	DPs 2, 6 and 8	[43]
*Pseudomonas elongate* MTCC5261	5.6/7.7	40	*K*_m_ = 6.66 mg/mL; *V*_max_ = 4 µmol min^−1^ mL^−1^	—	—	—	[45]
*Cytophaga*-like bacterium	7.2	40	*K*_m_ = 0.244 μM; *V*_max_ = 0.28 U	—	—	DP 2	[66]
*Vibrio* sp. CA-1004	8.0	40	*K*_m_ = 3.3 mg/mL	10% residual activity at 50 °C for 10 min	5.0 to 11.0	DP 2	[67]
*Cytophaga* MCA-2	7.2	—	—	—	—	DPs 4 and 6	[68]
*Tamlana* sp. HC4	8.0	30	*K*_m_ = 7.63 mg/mL	10% residual activity at 50 °C for 30 min	7.2 to 8.6	DPs 2 and 4	[52]
*Vibrio* sp. NJ-2	8.0	40	*K*_m_ = 2.54 mg/mL; *V*_max_ = 138.89 mmol mg^−1^ min^−1^	90% residual activity at 40 °C for 30 min	6.0 to 10.0	DPs 2–8	[54]
*Bacillus* sp. HT19	7.0	60	*K*_m_ = 0.061 mg/mL; *V*_max_ = 115.13 U/mg	90% residual activity at 60 °C for 24 h	—	DP 2	[69]
*Cellulophaga lytica* strain N5-2	7.0	35	*K*_m_ = 0.94 mg/mL; *V*_max_ = 13.42 U mg^−1^ min^−1^	75% residual activity at 35 °C for 2 h	5.0 to 8.0	DPs 6 and 8	[70]
*Pedobacter hainanensis* NJ-02	8.0	40	*K*_m_ = 2.4 mg/mL; *V*_max_ = 126 mmol mg^−1^ min^−1^	—	5.5 to 10.0	DPs 2, 4, 6 and 8	[71]
*Shewanella* sp. Kz7	8.0	45	*K*_m_ = 0.15 mg/L; *V*_max_ = 807.6 U/mg	85% residual activity at 30 °C for 1 h	pH 6.0 to 9.0	DPs 2 and 4	[72]
*Wenyingzhuangia aestuarii* OF219	8.0	25	*K*_m_ = 0.17 μM; *V*_max_ = 24.81 U/mg	42% residual activity at 25 °C for 24 h	pH 6.0 to 10.5	DPs 2 and 4	[73]
*Zobellia* sp. ZM-2	7.0	50	*K*_m_ = 2.07 mg/mL; *V*_max_ = 0.77 mg ml^−1^ min^−1^	89% residual activity at 55 °C for 10 min	—	DPs 4 and 6	[74]
*Zobellia* sp. ZL-4	7.0	55	*K*_m_ = 0.704 mg/mL	90% residual activity at 40 °C for 1 h	pH 5.0 to 8.0	DPs 2, 4, and 6	[75]
*Polaribacter* sp. NJDZ03	7.0	55	—	60–94% activity for 4–12 h at 55 °C	—	DP 2	[76]

Note: Original values are retained due to differing units reported in the source literature. “—” indicates data not reported in the original publication.

**Table 3:** Sources and biochemical properties of λ-carrageenases.

Organism	Optimal pH	Optimal Temperature (°C)	Thermostability	pH Stability	Hydrolysis Products	Reference
*Pseudoalteromonas carrageenovora* ATCC43555	7.5	30	—	—	DP 6	[77]
*Bacillus* sp. Lc50-1	8.0	75	50% residual activity after 85 °C for 10 min	6.0 to 9.0	DP 2	[46]
*Pseudoalteromonas* CL19	7.0	35	Stable at 25 °C for 120 min	—	DP 4	[78]

Note: Original values are retained due to differing units reported in the source literature. “—” indicates data not reported in the original publication.

**Table 4:** Sources and biochemical properties of ι-carrageenases.

Organism	Optimal pH	Optimal Temperature (°C)	Kinetic Parameters	Thermostability	pH Stability	Hydrolysis Products	Reference
*Zobellia galactanivorans*	—	—	—	—	—	—	[79]
*Microbulbifer thermotolerans* JAMB-A94^T^	7.5	50	*K*_m_ = 1.2 mg/mL	—	—	DP 4	[80]
*Wenyingzhuangia fucanilytica* CZ1127^T^	8.0	25	*K*_m_ = 1.12 μM; *k*_cat_ = 560.75 s^−1^	84% residual activity at 25 °C for 24 h	5.0 to 9.0	DP 4	[48]
*Cellulophaga* sp. QY3	7.0	50	*K*_m_ = 3.686 mM; *k*_cat_ = 14.226 s^−1^	80% residual activity at 50 °C for 1 h	More than 70% residual activity at pH 5.0–10.6 and 4 °C for 24 h	DPs 2 and 4	[81]
*Pseudoalteromonas carrageenovora* Psc^T^	8.3	40	*K*_m_ = 13.4 μM; *k*_cat_ = 1.64 min^−1^	—	—	—	[82]
*Flavobacterium* sp. YS-80-122	7.6	30	—	—	6.0 to 8.6	DP 4	[83]
*Pseudoalteromonas carrageenovora* ASY5	8	40	—	40% residual activity at 40 °C for 45 min	More than 60% residual activity at pH 8.0–9.5 and 4 °C for 75 min	DPs 2 and 4	[84]

Note: Original values are retained due to differing units reported in the source literature. “—” indicates data not reported in the original publication; “^T^” indicates the type strain of the species.

## 3. Structural Architecture, Substrate Recognition, and Catalytic Mechanisms of Carrageenases

Carrageenases generally exhibit a modular architecture consisting of catalytic domains (CDs) and carbohydrate-binding modules (CBMs), which collectively govern substrate specificity and catalytic efficiency [85]. CBMs anchor the enzyme to the glycan chain, aligning the scissile bond with the catalytic cleft to enhance processivity [86]. Additionally, positively charged residues within CBMs (e.g., Arg) form electrostatic interactions with the sulfate groups of carrageenan, further strengthening substrate binding and specificity [87]. The synergistic interplay between CDs and CBMs critically determines the degradation mode and the resulting oligosaccharide product profile. Consequently, a thorough understanding of this modular organization provides a rational basis for the molecular engineering of carrageenases with enhanced performance characteristics.

**Figure 3:**
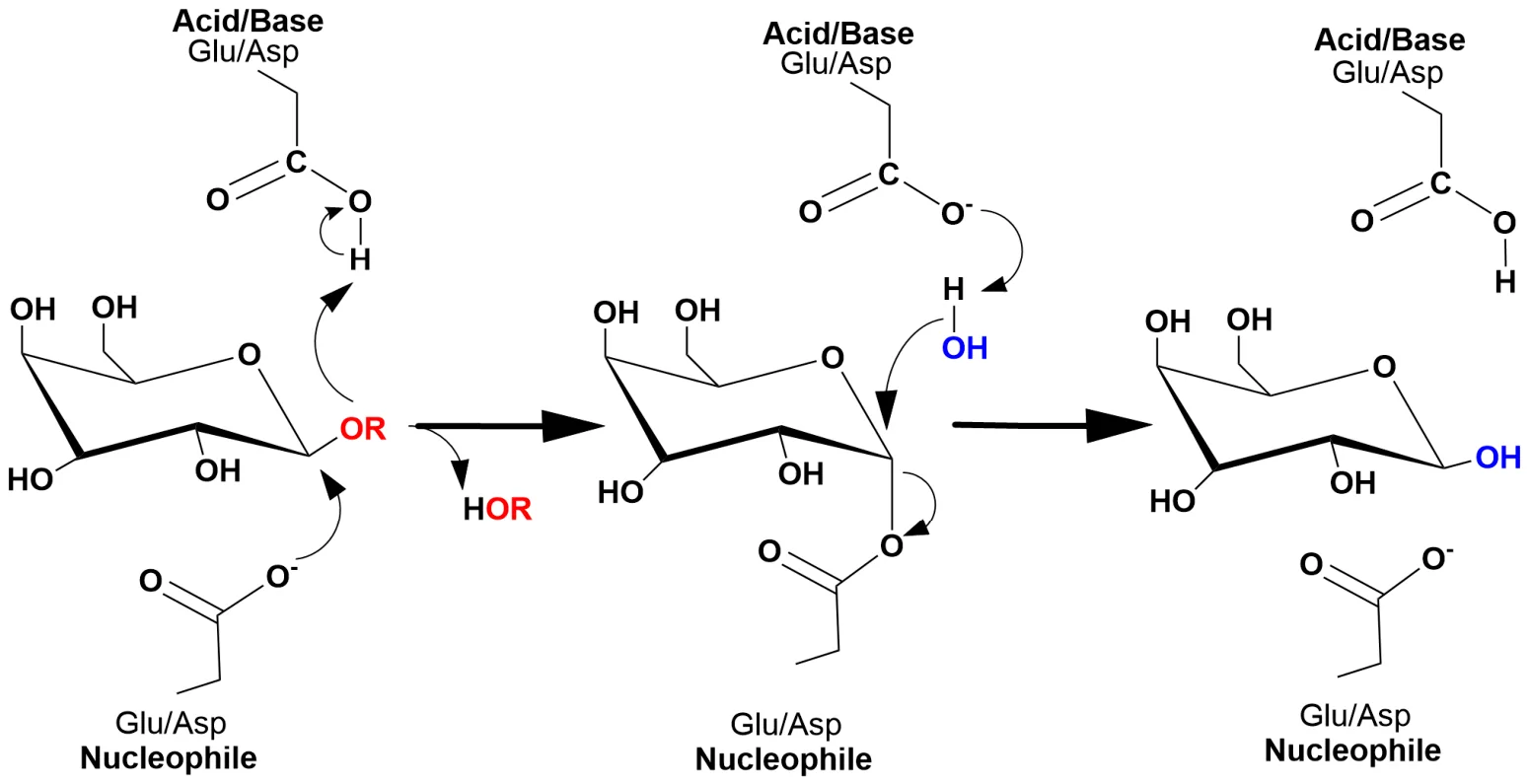
Retaining mechanism for the degradation of carrageenan by κ-carrageenase via double-displacement reaction [88]. Curved arrows indicate electron-pair migration, and straight horizontal arrows show the sequential progress of the reaction. Glutamate or aspartate residues function as the acid/base catalyst and nucleophile, respectively.

κ-Carrageenases belong to the GH16 family and adopt a typical β-jelly roll fold, consisting of two curved antiparallel β-sheets along with a small number of short β-strands and α-helices [36]. High-resolution structures from *Pseudoalteromonas carrageenovora* and *Zobellia* species reveal that the active site forms a cleft-like groove extending along the polysaccharide chain, supporting a processive cleavage mode [3,74]. Two strictly conserved glutamate residues within the E-X-D-E catalytic motif function as the nucleophile and the general acid/base, respectively, executing the classical GH16 retaining double-displacement mechanism (Figure 3) [88]. In this retaining mechanism, the enzyme catalyzes hydrolysis via a two-step reaction involving a covalent glycosyl-enzyme intermediate, with the anomeric configuration of the glycosidic bond retained in the final product [89]. The adjacent Asp facilitates catalysis by modulating the local microenvironment and stabilizing the transition state [36]. Variations in the loop regions at the termini of the catalytic channel and the “finger” structures among different κ-carrageenases, as well as the presence of non-catalytic domains such as the C-terminal PorS/Big_2 domain, significantly influence channel dimensions, substrate binding modes, and the resulting oligosaccharide product profiles [9]. κ-Carrageenases act in an endolytic manner on κ-carrageenan containing monosulfate groups, primarily producing κ-neocarrabiose (Nκ-2) and κ-neocarratetraose (Nκ-4) [49]. The prefix “neo” indicates that the non-reducing end of these oligosaccharides carries a 3,6-anhydrogalactose residue [4].

**Figure 4:**
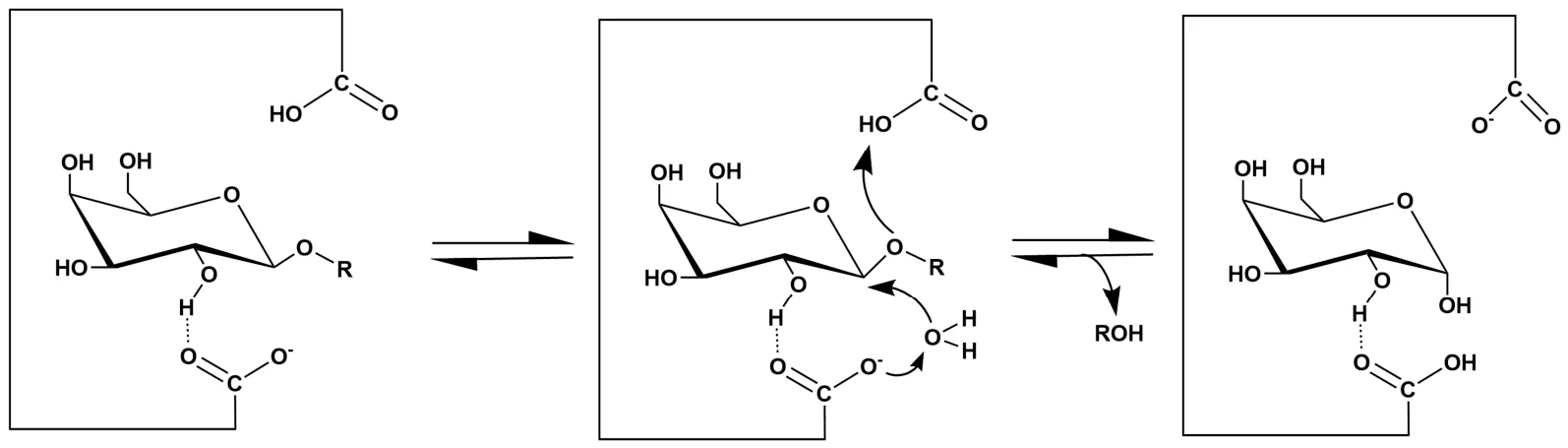
Inverting mechanism for the degradation of carrageenan by ι-carrageenase via single-displacement reaction [91]. Curved arrows indicate electron-pair migration, double-headed straight arrows denote reversible reaction equilibrium, and dashed lines represent hydrogen bonds.

In contrast, ι-carrageenases are classified into the GH82 family and feature a right-handed parallel β-helix as their main structural scaffold, with two additional domains at the C-terminus. Domain A adopts an α/β fold resembling a polyanion-binding protein [90]. Substrate binding models indicate that the tetrasaccharide substrate is anchored through interactions with multiple residues within the groove, and binding induces domain A to move toward the β-helix cleft, transitioning from an open conformation to a closed tunnel capable of accommodating the tetrasaccharide [31]. This conformational switching mechanism provides an explanation for the highly processive hydrolysis of polysaccharide chains employed by ι-carrageenases, contrasting with the processive mode of κ-carrageenases, which relies on fixed tunnels and loop regions [36]. The catalytic reaction of ι-carrageenase from *Alteromonas fortis* follows an inverting single-displacement mechanism (Figure 4) [91], with Glu245 serving as the proton donor and Asp247 acting as the general base to activate a water molecule for nucleophilic attack on the glycosidic bond [79]. Unlike the retaining mechanism, the inverting mechanism proceeds via a single-step direct displacement, in which a water molecule directly attacks the anomeric carbon, resulting in inversion of the anomeric configuration without formation of a covalent intermediate [89]. Other residues, such as His281 and Gln222, participate in substrate binding and stabilization of the catalytic water network, while Arg134 and Asn152 recognize the 2-sulfate group of the substrate via hydrogen bonding, ensuring specificity for ι-carrageenan [34]. The hydrolysis products being ι-neocarrabiose and ι-neocarratetraose, both of which exhibit immunomodulatory activity [31].

λ-Carrageenases belong to the GH150 family, and their three-dimensional structures have not yet been fully resolved [92]. Based on limited studies, they are predicted to adopt a (β/α)_8_-barrel fold and rely on divalent cations such as Ca^2+^ to alleviate the electrostatic repulsion and rigidity of the trisulfated λ-carrageenan chain, thereby facilitating hydrolysis [92]. In the λ-carrageenase mutant OUC-CglA, conserved tyrosine residues in the active center participate in sulfate coordination, while an aspartate serves as the catalytic acid/base or nucleophile [93]. Hydrolysis of λ-carrageenan yields λ-neocarrasaccharides (DP 2–8) with highly retained sulfate groups, whereas certain domain-truncated mutants preferentially generate longer-chain oligosaccharides of DP 10–20 [93]. These highly sulfated λ-carrageenan oligosaccharides and their derived nanomaterials exhibit application potential in areas such as anti-inflammation, antivirals, antitumor therapy, and drug delivery [94].

Despite significant progress in the structural characterization of κ- and ι-carrageenases, several bottlenecks remain. High-resolution structures are available for only a limited number of carrageenases, and no complete three-dimensional structure of a GH150 λ-carrageenase has been reported to date. Consequently, the substrate-binding mode and catalytic mechanism of λ-carrageenases remain largely speculative, which restricts rational design and functional optimization at the molecular level. The lack of structural insights into substrate recognition and cleavage of the densely sulfated backbone by GH150 enzymes critically constrains rational engineering efforts and precludes the systematic elucidation of structure–activity relationships for λ-carrageenan oligosaccharides. Furthermore, the conformational dynamics underlying the distinct processive versus non-processive degradation modes of different carrageenases are not fully understood. To accelerate structural elucidation, we advocate an integrated strategy combining high-throughput crystallization screening, small-angle X-ray scattering, cryo-electron microscopy, enhanced sampling molecular dynamics simulations, and Al-phaFold-guided prediction with experimental validation, thereby providing a more comprehensive framework for the engineering of carrageenases with tailored catalytic properties.

## 4. Molecular Engineering and Immobilization of Carrageenases

Carrageenases generally exhibit inferior thermostability compared with other in-dustrial polysaccharide-degrading enzymes. Many commercial cellulases retain over 80% activity after 2 h at 50 °C, with half-lives extending to 240 h under optimal conditions [95]. Thermostable α-amylases can maintain activity above 90 °C [96]. By contrast, most κ-carrageenases lose more than 50% of their activity within 1 h at temperatures exceeding 40 °C. Many are almost completely inactivated at 50 °C within the same period. This intrinsic instability arises from three primary degradation pathways. Thermal aggregation, proteolysis (including autoproteolysis), and oxidative inactivation are the major mechanisms. Among these, thermal aggregation appears to predominate. Elevated temperatures expose hydrophobic patches on the protein surface, leading to irreversible aggregation [97]. This process is particularly relevant for carrageenases, which possess flexible loop regions flanking the catalytic clefts. Successful thermostabilization strategies, including rigidification of finger regions, disulfide bond engineering, and non-catalytic domain fusion, primarily target conformational flexibility to prevent aggregation [98,99].

The inherent instability of carrageenases has driven extensive research into expressing truncated or mutated enzyme variants as well as immobilizing free enzymes onto solid supports [100]. Site-directed mutagenesis based on molecular dynamics (MD) simulations has identified key residues affecting thermostability. For κ-carrageenase from *P. tetraodonis*, Hong et al. predicted changes in folding free energy using the PoPMuSiC algorithm and screened the mutants Q42V and I51H via site-directed mutagenesis [101]. Mutants Q42V and I51H increased the enzyme activity by 20.9% and 25.4%, respectively, and elevated the *T*_m_ values by 7.0 °C and 3.6 °C, respectively, compared with those of the WT (51.2 °C) [101]. For κ-carrageenase from *P. porphyrae*, Du et al. used the PoPMuSiC algorithm combined with site-directed mutagenesis to obtain the E154A mutant, which showed a 29.4% increase in enzyme activity, a 51.6% higher residual activity than WT after 30 min at 50 °C, and a *T*_m_ elevated to 66.4 °C [102]. MD simulations revealed that enhancing the rigidity of the “finger region” and the flexibility of the catalytic pocket were key to the improved thermostability [102]. Sang et al. screened the S190R mutant of *P. porphyrae* κ-carrageenase using FoldX combined with MD simulations. The mutant, while retaining most of its activity, showed an increase in residual activity to 63.7% after 30 min at 50 °C, and its *T*_m_ rose from 64.4 °C to 66.2 °C [103]. For κ-carrageenase from *P. tetraodonis*, Zheng et al. screened two disulfide bond mutants of K44C-A119C and T120C-Q250C with improved enzyme activity and thermostability using the DbD2 strategy [104]. Wu et al. employed Discovery Studio to design the disulfide bond mutant N205C-G239C of *P. porphyrae* κ-carrageenase, which increased enzyme activity to 4.28 times that of WT, extended the half-life at 50 °C to 1.41 times, and achieved a residual activity of 82.5% after 30 min at 50 °C [105]. For κ-carrageenase from *P. porphyrae*, Zheng et al. obtained the D49I, Q100R, and the double mutant D49I-Q100R through surface charge engineering; the double mutant retained 91.0% of its initial activity after 7 days at pH 10.0, significantly outperforming the wild-type, demonstrating that surface charge remodeling can effectively enhance the alkali stability of κ-carrageenase [106]. Su et al. used surface charge engineering and consensus sequence analysis to mutagenize a κ-carrageenase from *P. tetraodonis* for improved alkaline tolerance [107]. Mutant D50N showed 1.25-fold wild-type catalytic activity and a 10.6% increase in residual activity (pH 9.0, 30 min); mutant E237R showed superior alkaline tolerance over the WT after 30 min, retaining 74.5% vs. 60.5% activity at pH 10.0, and 62.5% vs. 46.5% at pH 11.0 [107].

Non-catalytic domains can enhance the thermostability, substrate affinity, and processive hydrolysis capability of the carrageenases [98,108]. Furthermore, fusing non-catalytic domains (e.g., CBM, UNFD) from thermostable enzymes to the C-terminus of the target enzyme can greatly enhance its thermostability and substrate affinity. For example, after deletion of the C-terminal UNFD non-catalytic domain from the thermostable κ-carrageenase MtKC16A, the resulting truncated enzyme remained highly active at 100 °C without altering the oligosaccharide product profile [47,98]. Truncation of the C-terminal Por_Secre_tail (PorS) domain of the κ-carrageenase OUC-FaKC16A, although reducing its catalytic efficiency and thermal stability, unexpectedly enabled the resulting mutant OUC-FaKC16Q to hydrolyze κ-neocarratetraose (Nκ4) into κ-neocarrabiose (Nκ2) [109]. Studies show that among two κ-carrageenases from *Catenovulum agarivorans* DS2, CaKC16A (containing the nonCD domain) is superior to CaKC16B (lacking nonCD) in thermostability, substrate affinity, and catalytic efficiency, indicating that nonCD plays an important role in maintaining favorable enzyme characteristics [99]. Multiple studies indicate that improving thermostability primarily relies on enhancing the rigidity of the fingertip region and the flexibility of the catalytic pocket, whereas enhancing catalytic efficiency depends on optimizing non-catalytic domains to strengthen enzyme-substrate binding affinity.

In addition to genetic modification, enzyme immobilization represents another strategy to enhance reusability and tolerance to broader reaction conditions. To date, various carriers and methods, including magnetic nanoparticles, metal–organic frameworks (MOFs), and organic–inorganic hybrid materials, have been employed for carrageenase immobilization, significantly improving their catalytic performance and potential for industrial applications (Table 5). Studies have shown that κ-carrageenases from *Pseudoalteromonas* sp. ASY5 was successfully immobilized onto carboxyl-functionalized Fe_3_O_4_ nanoparticles, resulting in markedly enhanced pH stability and storage stability [84]. The catalytic domain of *P. porphyrae* κ-carrageenase was covalently immobilized onto amino-modified ZIF-8 and formed into cross-linked enzyme aggregates (CLEA), which retained high activity after multiple cycles of reuse [110]. The magnetic cross-linked nanoflowers (MCNF) of κ-carrageenase also exhibited excellent thermal/alkaline stability and reusability [111]. Broader reviews of enzyme-MOF and magnetic-MOF systems also indicate that MOFs offer advantages in enhancing thermal/chemical stability, enzyme loading capacity, and reusability through tunable pore sizes and surface functional groups [112]. Some studies employ organic nanomaterials (e.g., chitosan, alginate composites) or organic–inorganic hybrid materials to achieve enzyme immobilization via physical adsorption or entrapment, offering both biocompatibility and environmental friendliness [113,114]. The choice of immobilization strategy for carrageenases involves a trade-off among cost, performance, and scalability. Magnetic nanoparticles offer facile separation and reusability, yet their synthesis requires costly materials and complex functionalization [84]. Metal–organic frameworks (MOFs) provide high enzyme loading and enhanced thermal stability due to their tunable porosity. However, their scalable synthesis and long-term operational stability remain challenging for industrial use [113]. Sodium alginate beads are inexpensive and simple to prepare, but their dense gel network imposes mass transfer resistance, reducing apparent catalytic efficiency. For high-value pharmaceutical products, the superior performance of nanoparticle- or MOF-based systems may justify higher costs. For bulk applications such as feed additives, the cost-effectiveness of alginate-based carriers renders them more competitive. Future efforts should focus on developing cost-effective synthetic routes for advanced carriers and engineering hydrogel porosity to alleviate diffusion limitations.

From a process-economic perspective, the feasibility of enzymatic carrageenan processing is governed by enzyme loading and recyclability, substrate concentration, volumetric productivity, target-product yield, and downstream recovery. Compared with chemical depolymerization, enzymatic conversion shifts a greater proportion of the operating expenditure toward biocatalyst production and reaction time. Nevertheless, its mild reaction conditions and greater control over the degree of polymerization and sulfation pattern can reduce product heterogeneity and the subsequent purification burden [22]. It has been reported that hydrolysis of 5 g of κ-carrageenan using 40 mg of commercial α-amylase afforded a product yield of 92.6% [117]. This activity is attributed to the intrinsic multifunctionality or substrate promiscuity of certain α-amylases, which have been reported to possess additional carrageenase, agarase, and cellulase activities [118,119]. Direct two-step treatment of *Kappaphycus alvarezii* (formerly *Eucheuma cottonii*) with cellulase and recombinant κ-carrageenase achieved an oligosaccharide recovery of 38% [120], whereas microwave-assisted mild acid hydrolysis yielded 22.9 ± 0.8% target disaccharide despite an overall sugar recovery of approximately 90% [121]. These results suggest that enzymatic processing is currently more attractive for structurally defined, high-value oligosaccharides, with further cost reductions expected from high-titer enzyme production, immobilization and reuse, increased substrate loading, and continuous or hybrid processing.

## 5. Applications and Prospects of Carrageenases and Their Enzymatic Hydrolysis Products

As specific glycoside hydrolases, carrageenases demonstrate transformative potential in industrial and biomedical fields. In agricultural applications, oligosaccharides derived from the novel κ-carrageenase CgkA serve as feed additives that enhance growth and immune responses in crucian carp [122]. Similarly, ι-carrageenase is employed in dairy processing to prevent gel syneresis, thereby enhancing the textural stability of low-fat yogurt [123]. It has been reported that Cg82Mf, a GH82 family random endo-ι-carrageenase, also exhibits hydrolytic activity toward κ-carrageenan, indicating its potential as a biocatalyst for carrageenan tailoring [34]. Research has shown that bacterial κ-carrageenase from *Pseudomonas elongata* MTCC 5168 is essential for the efficient isolation of protoplasts from the red alga *Kappaphycus alvarezii* when used in combination with cellulase and macerozyme, as no protoplasts could be obtained in the absence of the carrageenase [124]. In environmental remediation, engineered κ-carrageenases with enhanced thermostability or alkali tolerance have been applied to the valorization of industrial κ-carrageenan waste residues [105]. Carrageenases are also utilized in environmental remediation, such as enzymatic hydrolysis of algal polysaccharides in aquaculture wastewater, effectively lowering chemical oxygen demand and mitigating eutrophication [125].

**Figure 5:**
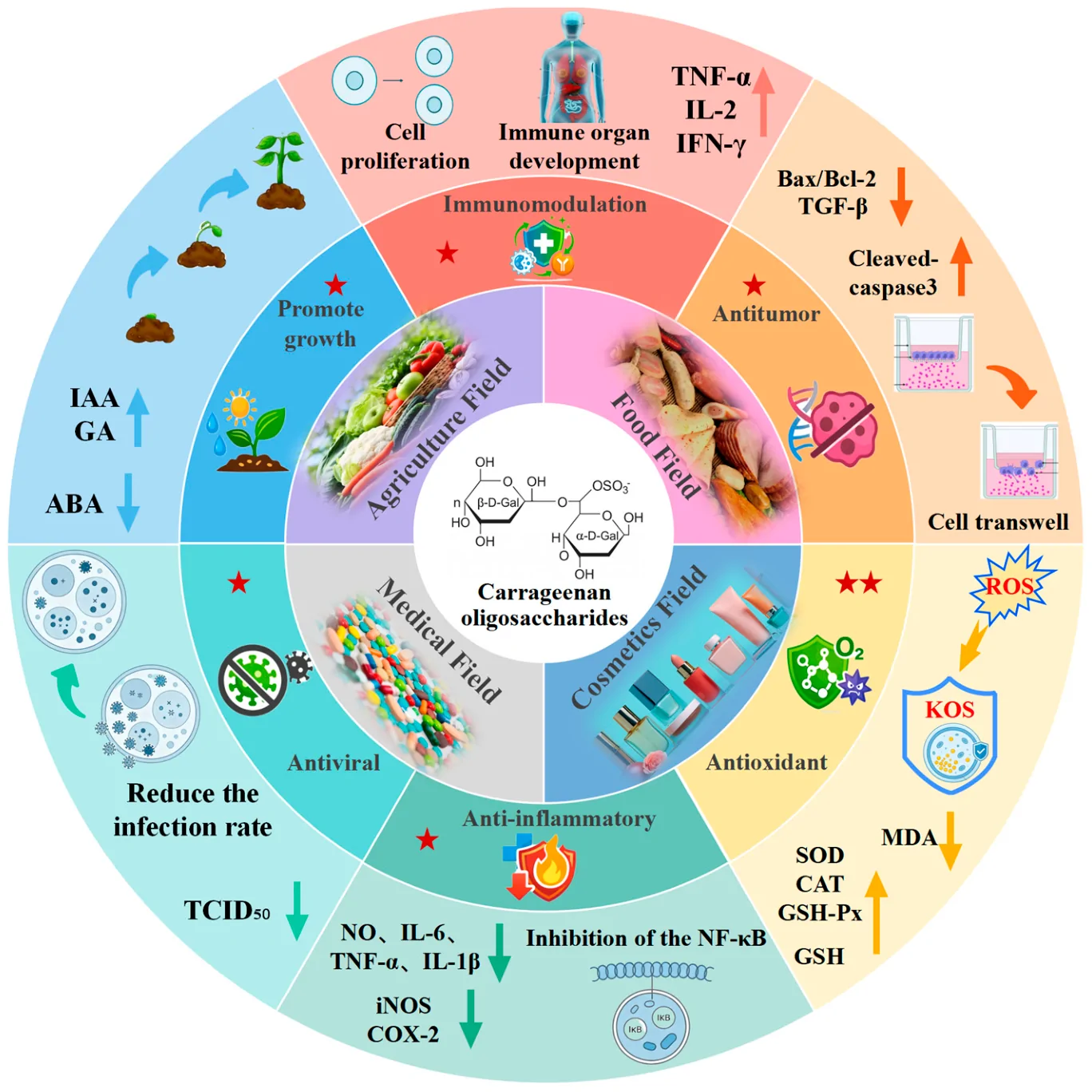
Bioactivities and application fields of carrageenan hydrolysates. The quantity of asterisks (★) indicates the maturity of research and industrial transformation for each field. Upward arrows (↑) indicate the upregulation or increase of related indicators; downward arrows (↓) represent the downregulation or decrease of related indicators.

In the biomedical field, κ-carrageenan oligosaccharides hold great application potential, owing to their low molecular weight and the structure–activity relationships driven by sulfate groups. Recent studies on the biological activities of carrageenan oligosaccharides are illustrated in Figure 5, which summarizes the major bioactivities and application fields reported to date. Recent studies show that oligomeric configurations such as κ-carrabiose and κ-carratetraose possess broad-spectrum inhibitory potential against various enveloped viruses [126]. In an in vitro hyperlipidemic cell model, κ-carrageenan oligosaccharides exert hypolipidemic effects by activating the AMPK pathway and downregulating lipogenic proteins, thereby providing new insights for functional lipid-lowering ingredients [110]. In mouse tumor models, λ-carrageenan oligosaccharides enhance macrophage phagocytic function, promote the secretion of cytokines such as TNF-α and IFN-γ, and increase the infiltration of M1-type macrophages and activated T cells within the tumor microenvironment, thereby boosting antitumor immune responses [122,127]. Moreover, the combination of λ-carrageenan oligosaccharides with 5-fluorouracil (5-FU) synergistically enhances anti-gastric cancer activity and alleviates the immunosuppression induced by 5-FU monotherapy, demonstrating their potential as immunomodulatory adjuvants [128]. Furthermore, carrageenan oligosaccharide-based nanoparticles (e.g., chitosan/κ-oligosaccharide complexes) achieve high drug loading and sustained release under the mildly acidic tumor microenvironment, enhancing the efficacy of tumor-targeted therapy [129]. κ-Carrageenan oligosaccharides can directly scavenge hydroxyl radicals, superoxide anions, and DPPH radicals, thereby blocking free radical chain reactions; oligosaccharides with low molecular weight and high sulfation degree exhibit stronger scavenging capacity [130]. κ-Carrageenan oligosaccharides chelate Fe^2+^ and Cu^2+^ to suppress Fenton reaction and hydroxyl radical generation, while upregulating hydration- and barrier-related proteins, thereby attenuating PM2.5-triggered oxidative stress, inflammation, hydration deficiency, and barrier disruption in HaCaT cells [131]. In cellular and animal models, intervention with κ-carrageenan oligosaccharides significantly increases the activities of superoxide dismutase and catalase, reduces malondialdehyde levels, and effectively alleviates oxidative stress damage [132,133]. Mechanistic studies indicate that κ-carrageenan oligosaccharides activate the Nrf2/ARE signaling pathway, upregulate the expression of antioxidant genes, and inhibit the overactivation of oxidative stress-related pathways such as MAPK and NF-κB, thereby exerting a comprehensive antioxidant protective effect [134]. Research has shown that κ-carrageenan oligosaccharides significantly downregulate the expression of pro-inflammatory factors including COX-2, IL-6, and TNF-α, and inhibit classic inflammatory signaling pathways such as NF-κB, MAPK, and TLR4, demonstrating clear anti-inflammatory activity [135,136]. In a colitis model, treatment with κ-carrageenan oligosaccharides upregulates the IL-10 expression, reduces the epithelial cell apoptosis, and repairs the intestinal barrier integrity [137]. Moreover, κ-carrageenan oligosaccharides can modulate the gut microbiota composition, increasing the abundance of beneficial bacteria (e.g., *Lachnospiraceae*) and decreasing the abundance of pathogenic bacteria (e.g., *Bacteroides*), synergistically alleviating chronic inflammation [138]. Furthermore, ι-carrageenan oligosaccharides ameliorate DSS-induced colitis in mice by modulating gut microbiota dysbiosis and regulating the SCFAs-PI3K-AKT pathway [139]. Notably, certain gut microbiota can metabolize low-molecular-weight κ/ι-carrageenan oligosaccharides into pro-inflammatory substances, highlighting the need for structural optimization and safety evaluation in application development [140].

Carrageenan (high-molecular-weight) is permitted as a food additive (thicken-er/stabilizer, E 407) in the United States (GRAS, 21 CFR 182.7255), the European Union (Regulation (EC) No 1333/2008), and China (GB 2760). By contrast, carrageenan oligo-saccharides lack regulatory authorization as food additives or conventional food ingredients in these jurisdictions. Degraded carrageenan (poligeenan), produced by acid hydrolysis, is prohibited in food systems and should not be conflated with enzymatically prepared oligosaccharides. Nevertheless, the gastrointestinal effects of low-molecular-weight fragments remain a core safety concern requiring thorough evaluation [137].

**Figure 6:**
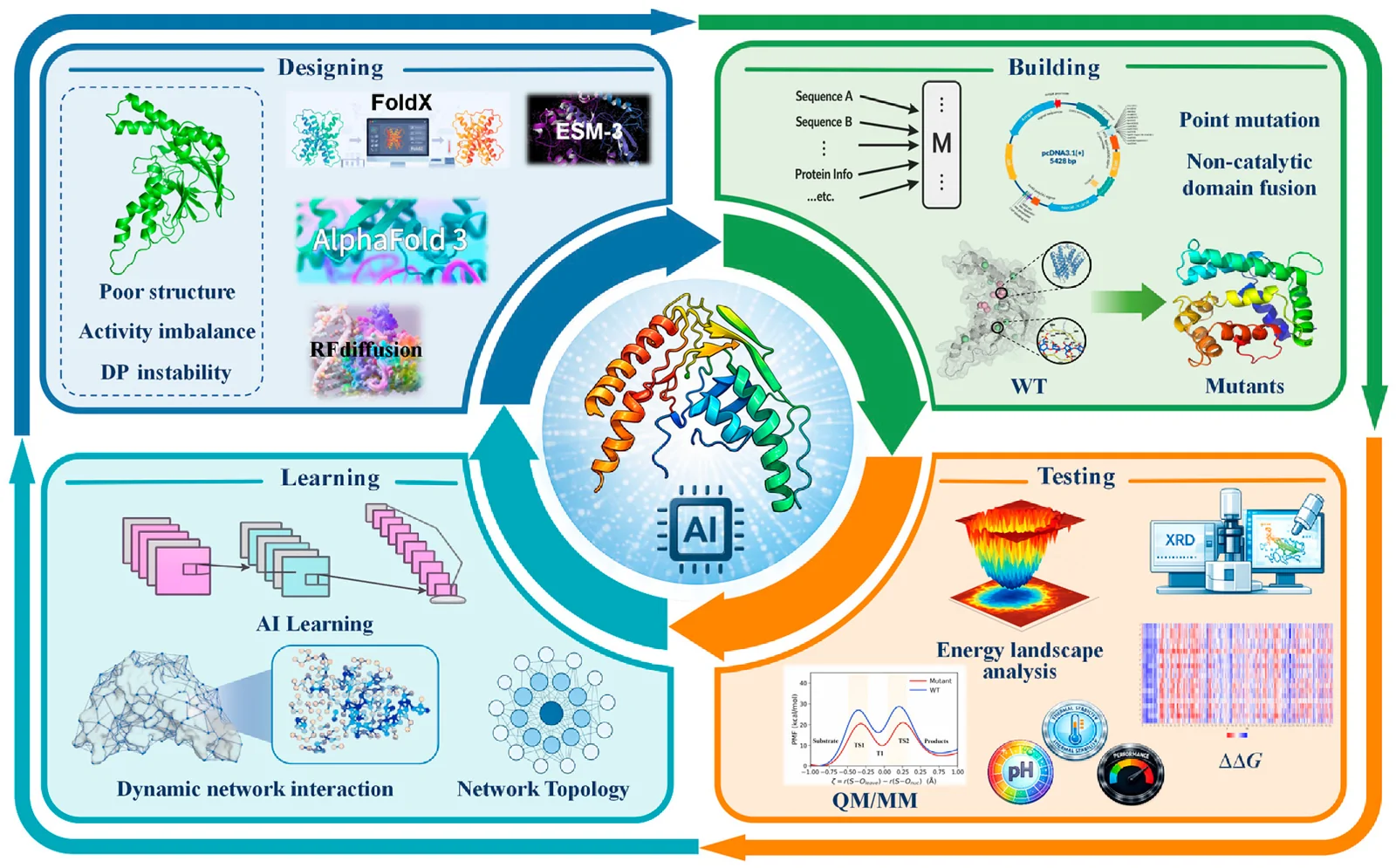
The “Designing–Building–Testing–Learning” cycle for carrageenase engineering. Colored thick arrows indicate the iterative workflow among modules.

Although conventional molecular engineering strategies have significantly improved the industrial performance of carrageenases, achieving synergistic optimization of multiple enzyme properties (e.g., activity, stability, and specificity) remains a major challenge in the complex conversion from seaweed biomass to high-value oligosaccharides. In recent years, the rise in artificial intelligence (AI) has provided a new paradigm to overcome this bottleneck. In carrageenase research, AI technologies have begun to be applied to elucidate the catalytic mechanisms and guide the molecular engineering. The “Designing–Building–Testing–Learning” approach for carrageenases is illustrated in Figure 6, which depicts how AI-assisted tools are integrated into each stage of the engineering pipeline. Studies have shown that attention mechanism-based protein language models can automatically extract functional “protein vocabularies” from sequence representations [141], such models can be used to autonomously identify key functional residues in carrageenase sequences, establishing a mapping between sequence segments and catalytic activity. Computational strategies incorporating conformation-biased mutations indicate that modulating the subtle energy distribution within conformational landscapes can effectively tune protein function [142], enabling precise regulation of the flexibility of the carrageenase catalytic pocket while simultaneously enhancing both thermostability and catalytic efficiency. Inverse folding frameworks that integrate structural and evolutionary information further improve the generalization of sequence–structure–function relationships [143], facilitating the design of carrageenase mutants with specific substrate selectivity and expanding their catalytic spectrum toward κ-, ι-, and λ-carrageenans. Additionally, cross-molecular-modal binding site recognition can be applied to predict the interaction interfaces between carrageenases and their substrates [144], while geometry-based enzyme retrieval methods provide efficient tools for discovering novel carrageenase genes [145]. These approaches demonstrate that modern AI models are increasingly capable of capturing cooperative residue interactions in carrageenases, offering new possibilities to overcome the limitations of traditional mutagenesis strategies.

Recent studies have demonstrated the tangible integration of AI tools into carrageenase discovery and engineering. Liu et al. developed ETopt, a multimodal machine learning model that predicts the optimal catalytic temperature (T_opt_) of carbohydrate-active enzymes, and successfully applied it to identify thermophilic ι-carrageenases suitable for high-temperature bioprocessing of gelling marine polysaccharides [146]. Zheng et al. employed AlphaFold2 to identify Cg82Mf, a GH82 family protein predicted to lack a lid structure on the catalytic groove; experimental validation confirmed it as the first reported random endo-acting ι-carrageenase with bifunctional activity against both ι- and κ-carrageenan [34]. Vickers et al. used X-ray crystallography combined with Al-phaFold2-predicted models to elucidate the complete λ-carrageenan depolymerization cascade from *Pseudoalteromonas distincta* U2A, revealing a sophisticated cyclic pathway involving ten glycoside hydrolases and five sulfatases [92]. Tingley et al. applied AlphaFold3 to interpret the structural basis of substrate specificity within GH16_17 carrageenases, demonstrating how AI-generated structural models can explain the functional diversity of carrageenan-degrading enzymes identified through functional microbiome analyses of ruminant gut microbiomes [147]. Collectively, these examples illustrate that AI tools are transitioning from speculative prediction to experimental validation, enabling the discovery of enzymes with previously unreported activities and providing structural insights that were unattainable through conventional methods alone.

Concurrent advances in generative modeling and structure-guided optimization are accelerating the transition of carrageenase research from empirical engineering to rational design. Frameworks based on diffusion architectures have achieved de novo construction at atomic precision, demonstrating the feasibility of high-accuracy macromolecular generation [148], which can facilitate the de novo design of carrageenase catalytic domains. Hydrogen bond network maximization strategies have enabled the computational design of ultra-stable carrageenases, highlighting the critical role of fine-grained physicochemical optimization [149]. Differential equation-based dynamic models facilitate the continuous characterization of the binding process between carrageenases and their substrates, enhancing the interpretability of interaction kinetics [150]. Structure-guided mutational scanning combined with iterative screening further exemplifies how the integration of predictive modeling and experimental screening can synergistically enhance both catalytic efficiency and stability of carrageenases [151]. These advances mark a transition from empirical mutagenesis to model-driven, multi-objective synergistic optimization. Future breakthroughs may integrate AI-driven enzyme design with circular bioeconomy models, incorporating multi-product algal biorefinery processes combined with AI-driven process optimization to achieve the synergistic production of high-value carrageenan oligosaccharides and biofertilizers, thereby reducing processing costs and enhancing economic and environmental benefits [152]. Moreover, improving the prediction and decision-making efficiency of AI algorithms in enzyme molecular design, carrageenase fermentation optimization, and waste valorization will promote the intelligent and green development of carrageenase research and applications.

## 6. Conclusions and Outlook

In summary, carrageenase research has gradually transitioned from enzyme discovery and characterization to a new phase of synergistic advancement in structural resolution, performance optimization, and application translation. Although systematic understanding has been achieved for κ- and ι-carrageenases regarding catalytic mechanisms and structure–function relationships, significant gaps remain for λ-carrageenases, particularly the GH150 family, in terms of structural basis, catalytic nature, and fine substrate recognition. Therefore, the key challenge in this field is achieving multi-objective synergistic optimization centered on activity, stability, specificity, product controllability, and process compatibility. To this end, molecular engineering and immobilization technologies have advanced the application development of carrageenases at the levels of intrinsic enzyme properties and process operational performance, respectively. However, industrialization remains constrained by practical issues such as the activity–stability trade-off, mass transfer limitations, cost control, and scale-up consistency. Meanwhile, carrageenan oligosaccharides show promising prospects in food processing, functional materials, and bioactive development. Nevertheless, their antioxidant, anti-inflammatory, antitumor, and antiviral activities are currently primarily based on in vitro experiments and animal models. More systematic studies are needed concerning structure–activity relationships, mechanisms of action, safety evaluation, and standardized preparation. As optimization goals shift from single-property enhancement to multi-parameter synergistic design, traditional empirical engineering strategies are no longer sufficient. Thus, artificial intelligence is not merely a technical supplement but a critical tool to drive carrageenase research from experience-driven to prediction-driven and rationally designed approaches. Future efforts should further integrate structural biology, enzyme engineering, high-throughput experimentation, and data-driven methods to establish a closed-loop “Designing–Building–Testing–Learning” optimization system, thereby providing a more solid theoretical foundation and technical support for the in-depth application of carrageenases in green manufacturing of high-value oligosaccharides and related biomedical developments.

## Figures and Tables

**Table 5:** Comparative analysis of κ-carrageenases immobilization strategies.

Support Material	Immobilization Method	Enzyme Loading/Immobilization Efficiency	Optimum pH/Temperature	Reusability	Thermal/Storage Stability	Reference
Carboxyl-functionalized Fe_3_O_4_ nanoparticles	Covalent binding	326.0 U·g^−1^ support; enzyme recovery 46.9%	pH 7.5; free enzyme 60 °C, immobilized enzyme 55 °C	43.5% residual activity after 4 cycles	—	[84]
Magnetic Fe_3_O_4_-chitosan microspheres	Entrapment (immobilized bacteria)	—	—	≥7 cycles	—	[115]
Amine-modified ZIF-8 + CLEA	Covalent immobilization	—	—	—	—	[110]
PEI-modified pomelo peel cellulose	Physical adsorption	Enzyme activity recovery >50% (4.1-fold of unmodified carrier)	pH 7.5; 60 °C (same as free enzyme)	46.8% activity loss after 3 cycles	Decreased thermal tolerance; improved pH tolerance; 76.0% activity retained after 98 days of storage	[40]
Inorganic hybrid nanoflowers (CaNF@CgkPZ)	Self-assembly	Kcat/Km 382.1 mL·mg^−1^·s^−1^, 292% higher than free enzyme	—	—	70–100% relative activity retained after 15 days of storage at 4–25 °C	[116]
Magnetic cross-linked nanoflowers (MCNF)	Synergistic integration (enzyme + CaP + MNPs)	—	—	—	Enhanced thermal stability at 45–55 °C; enhanced stability at pH 6.0 and 12.0	[22]

Note: “—” indicates data not reported in the original publication.

## Data Availability

No new data were created or analyzed in this study. Data sharing is not applicable to this article.
